# Fractal Modeling of Polymer Plasma Laser Ablation, Plasma Plume Tsallis Entropy and Its q-Statistics Interpretation, Part I: Theory

**DOI:** 10.3390/e24030342

**Published:** 2022-02-27

**Authors:** Maria-Alexandra Paun, Vladimir-Alexandru Paun, Viorel-Puiu Paun

**Affiliations:** 1 School of Engineering, Swiss Federal Institute of Technology (EPFL), 1015 Lausanne, Switzerland; maria-alexandra.paun@epfl.ch; 2 Division Radio Monitoring and Equipment, Section Market Access and Conformity, Federal Office of Communications OFCOM, Federal Department of the Environment, Transport, Energy and Communications DETEC, Rue de l’Avenir 44, 2501 Bienne, Switzerland; 3 Five Rescue Research Laboratory, 75004 Paris, France; vladimir.alexandru.paun@ieee.org; 4 Department of Physics, Faculty of Applied Sciences, University Politehnica of Bucharest, 060042 Bucharest, Romania; 5 Academy of Romanian Scientists, 050094 Bucharest, Romania

**Keywords:** fractal space-time, Tsallis entropy, polymer, plasma, laser ablation

## Abstract

Polymer plasma produced by laser ablation is investigated in a theoretical manner. In relation to the fact that the charge carrier circulation is assumed to take place on fractal curves, the so-called fractality type, electrical charge transport can be resolved by an extended scale relativity method. In addition, an elegant mathematical model, utilizing a conjecture of fractal space-time, is elaborated. The complete solution and its graphical representation for temperature distribution in two-dimensional and three-dimensional cases are successfully introduced. The discrete physical behavior and irrevocable transformation of nanoscale microdomain substructures by laser ablation are realistically examined. Further, benefiting from the interpretation of the fractal analysis, each of the experimental results can be fairly explained. On top of that, this paper presents a proof of Tsallis nonextensive q-statistics, especially for the plasma plume studied. Tsallis entropy in direct connection with fractal dynamics and chaotic-type mechanics of the plasma plume and time-series representation of plasma temperature is introduced for the first time in the present publication, and the q-statistics of the plume plasma temperature are also studied, among others.

## 1. Introduction

This article has its origins in some fundamental studies on transient plasmas generated by laser ablation in different time regimes (nanosecond (ns), picosecond (ps) and femtosecond (fs)), on targets made of polymeric materials and advanced theoretical investigations using a mathematical fractal model to study the dynamics of so-called ablation plasmas. The basic concept of laser ablation is straightforward and refers to a laser pulse that is focused on the surface of a target in the state of solid aggregation, which leads to the transformation of a microvolume into a gas phase and the formation of a structure with several components, in which atoms, ions, molecules, clusters, nanoparticles, electrons and photons can coexist. This structure will evolve further through expansion in vacuum or ambient gas in various forms, as well as spatial and temporal extensions dependent on the experimental conditions imposed from the outside.

A relatively simple way to describe the laser ablation process in detail is to split it into four main steps. Partial or total absorption of the energy of the laser beam induces an increase in temperature, followed by the diffusion of heat into the material. This heating can cause a phase transition or chemical degradation of the thermally activated system (depending on the wavelength used, photochemical processes can also occur, especially in the UV field). The ejection of matter in the form of atoms, molecules and clusters occurs if the temperature is high enough. The rapid expansion of matter (with speeds generally in the order of tens of km/s) leads to the creation of a shock wave. Depending on the duration of the pulse, this step may be followed by the absorption of incident radiation (for laser pulses longer than 10 ps). The ejected matter can interact with the laser pulse (≫10 s) by diffusion, absorption, etc. This forms and develops a plasma with transient properties on the surface of the irradiated material. After the laser pulse is completed, the material begins to cool, and the ionized species recombines. Some of the ejected matter may recondense and redeposit on the surface.

A recent method of examining the dynamics of plasma is to take into account that the motions of electrically loaded particles occur in continuous curves (or continuous only on portions of them) but are considered to be essentially nondifferentiable (at the same time), i.e., on fractal-type trajectories [1,2,3,4,5,6,7]. Subsequently, the complex comportment of these dynamic systems is theoretically replaced by the fractality idea, both as breaking/rupture lines in alloys subjected to mechanical testing [5] and as curves on which it travels through electrical charge transport, all being considered mathematically nondifferentiable curves/trajectories [6].

The fractal space-time theory is considered a serious ‘test of the mind’, in which moving corpuscles are restricted to movement on fractal-type trajectories, which brings into discussion the Peano–Moore path case in particular [8].

Given the remarkable physical and chemical properties of polymeric materials, polymeric plasmas were successfully obtained by laser ablation procedure. Thus, among the first experiments that used polymeric materials subjected to a far-ultraviolet excimer laser radiation as targets, we can cite Reference [9].

The present work is organized into four sections. After the introductory remarks, the second chapter is devoted to materials and methods, with an emphasis on the mathematical model in the fractal conjecture of space-time physics relying on solid mathematical apparatus. At this point, Tsallis q-statistics are also interpreted, and the experimental technique is presented. Section 3, titled Results and Discussion, first introduces the theoretical results, with an analysis of various aspects, including plasma expansion, transient ionic current and temporal ion current oscillation, as well as the two-dimensional time and position evolution of temperature. The last section, Section 4, presents the conclusions. In addition, for the first time, it is announced that Tsallis entropy has a direct connection with fractal dynamics and chaotic-type mechanics of plume plasmas.

## 2. Materials and Methods

In the current article, a theoretical examination of the laser ablation of polymeric materials is conducted. The present review depicts only the physical comportment (behavior) of polymer plasma plume produced through the laser ablation process, without considering at all the chemical reactions that can take place at the same time through the thermal regime of the material support suffered [10,11,12].

Let us present in a few words the physical phenomena that take place in the laser ablation procedure and obtain the so-called plasma plume structure. Consequently, the heating process determines material melting or vaporization, thereby establishing the dismissal of the visible background mass from the specific basic zone of the sample used. The transition from the solid aggregation state into the gas aggregation state has repercussions on plasma plume formation. In other words, ablation is a conjunction between the two processes that occur instantly, the first being vaporization and the second being melt expulsion, naturally [13].

**Figure 1:**
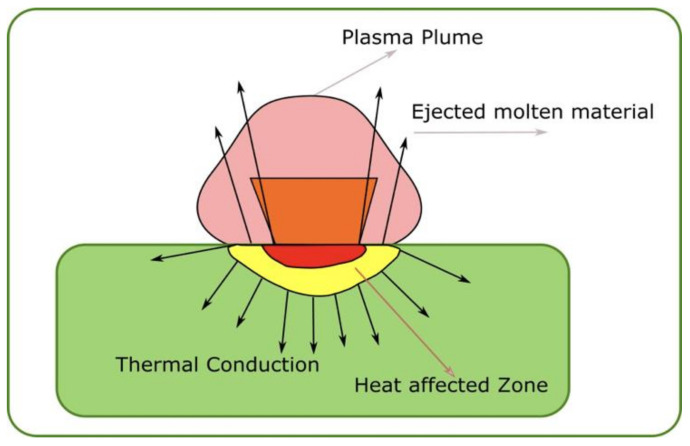
Simplified block diagram of laser–material interface.

In Figure 1, the simplified block diagram of the laser–material interface is drawn. Here are found the most important areas, such as entitled plasma plume and heat-affected zones closely related to the thermal conduction zone. These areas are well demarcated and colored differently, e.g., the plasma plume area has a dark pink color, the thermal conduction area has a green color and the heat-affected area has a yellow color.

In this complex process, the input data are determinants that are related to the type of laser used, among which we mention pulse duration, wavelength, laser power and repetition rate, but also to the optical setup, which does not lack fluence, pulse number and transition speed.

As an expected result, called outputs, we name the following requirements: aspect ratio, redeposition, heat-affected zone and crater formation.

We must now make, with the beginning statement, the assertion that this paper is primarily theoretical.

### 2.1. Mathematical Model in the Fractal Space-Time Theory

#### 2.1.1. Fractal Calculation

Historically speaking, we can say that the fractional calculation was initiated only in Newton’s epoch in its incipient aspects, and in the present period, it has matured into the most fervent subject in different scientific areas, with significant developments happening even today.

Ab initio, we can say that everything starts from the mathematical definition of the speed of an object, which is still retained (in the same concept) in the theory of fractional calculus, albeit with a different presentation specific to the new theory.

Likewise, the new expression for velocity in the fractal space-time configuration (*x^β^*, *t^α^*) can be reconsidered and redefined as follows

(1) $$v^{\prime }=\frac{dx^{\prime }}{dt^{\prime }}=\frac{dx^{\beta }}{dt^{\alpha }},\alpha ,\beta \geq 0$$

In the formula above, $S^{\alpha ,\beta }$ describes the fractal space-time configuration, when *α* and *β* are indices of scale [14].

##### Definitions

Consistent with the debate previously presented, the fractal-type derivative notion of a function *u*(*t*), reported to a fractal time-scale dimension *t*, is now

(2) $$\frac{\partial^{}}{\partial t^{\alpha }}\left[f(t)\right]=\underset{t_{1}\to t}{\lim}\frac{f(t_{1})-f(t_{})}{t_{1}^{\alpha }-t^{\alpha }},\alpha \geq 0.$$

The most general definition becomes the following

(3) $$\frac{\partial^{\beta }f(t)}{\partial t^{\alpha }}=\underset{t_{1}\to t}{\lim}\frac{f^{\beta }(t_{1})-f^{\beta }(t)}{t_{1}^{\alpha }-t^{\alpha }},\alpha \geq 0\;and\;\beta \geq 0.$$

##### Connection with Derivative

One point that can be presented again is that the fractal-type derivation (a classical Hausdorff derivation, in the subsidiary) in relation to time is as presented in Formula (3). It is worth mentioning here the fact that still exists a few more definitions for fractal-type derivation, known well in the literature.

When both the *Df* derivation operator and the *Dαf* fractal derivation operator exist simultaneously, there is parallelism between the two, based on the classic chain rule

(4) $$\frac{\partial^{}f}{\partial x^{\alpha }}=\frac{df}{dx}\frac{dx}{dx^{\alpha }}=\frac{1}{\alpha }x^{1-\alpha }\frac{df}{dx}$$

The next level of calculation, conforming to the theorem of implicit function, leads, under adequate circumstances, to $\frac{dx}{dx^{\alpha }}={\left(\frac{dx^{\alpha }}{dx}\right)}^{-1}$.

Analogically, the extensive definition we have at our disposal is

(5) $$\frac{\partial^{\beta }f}{\partial^{\alpha }x}=\frac{d(f^{\beta })}{d^{\alpha }x}=\frac{1}{\alpha }x^{1-\alpha }\beta f^{\beta -1}(x)f^{\prime }(x).$$

*F^α^-calculus, F-limit and F-continuity*.

We now establish the rules of the limit and continuity procedure over a fractal-type curve called F-limit and F-continuity.

Let F be a curve in an m-dimensional space, or more precisely, m is the embedding dimension, inasmuch as $F\subset R^{n}$ is a curve [15].

**Definition** **1.** *Let* $F\subset R^{n}$ *be a fractal curve, and let* f:F→R. *A number l is said to be the limit of f through points of F, or simply F-limit, as* $\theta^{\prime }\to \theta ,$ *. If* ε≥0 *is given, there exists* δ≥0 *such that*

(6) $$\theta^{\prime }\in F\;and\;\left|\theta^{\prime }-\theta \right|\leq \delta \Rightarrow \left|f(\theta^{\prime })-l\right|\leq \varepsilon .$$

In the case in which the number *l* exists, it will be indicated as having the value

(7) $$l=F-\underset{\theta^{\prime }\to \theta }{\lim}f(\theta^{\prime }).$$

**Definition** **2.** *A function* f:F→R *is said to be F-continuous at* θ∈F, *if*

(8) $$f(\theta )=F-\underset{\theta^{\prime }-\theta }{\lim}f(\theta^{\prime }).$$

**Definition** **3.** f:F→R*is said to be uniformly continuous on* E⊂F,*if for any*ε≥0*there exists*δ≥0*such that for any*θ∈F*and*$\theta^{\prime }\in E$

(9) $$\left|\theta^{\prime }-\theta \right|\leq \delta \Rightarrow \left|f(\theta^{\prime })-l\right|\leq \varepsilon .$$

#### 2.1.2. Fractal Space-Time Theory

Suppose now that the movement of electrically loaded particles occurs on continuous curves (or continuous only on portions of them) but is considered to be essentially nondifferentiable (at the same time), i.e., on fractals [7]. A geometrical curve that bends and curls at every level of magnification is a fractal curve. It has a fractional dimension between 1 and 2. Consequently, a fractal curve is a rectifiable curve. The fractal dimension notion can be examined for different fractal-type curves or dust that are not self-similar but consider certain diagonally self-affine fractals obtained by a recursive cascade (see Reference [6]).

The nondifferentiability concept from the topological space with fractal dimension $D_{F}$ assumes a replacement of the ordinary time derivative operator d/dt by an unusual complex operator $\frac{\hat{d}}{dt}$

(10) $$\frac{\hat{d}}{dt}=\frac{\partial }{\partial t}+V_{C}\cdot \nabla -i\frac{\lambda^{2}}{2\tau }{\left(\frac{dt}{\tau }\right)}^{\left(\frac{2}{D_{F}}\right)-1}\Delta$$

where V_c_ is the complex velocity, Vc=V+iU. In Formula (10), dt is the time resolution, τ is the fractal–nonfractal transition time and λ is the characteristic length scale. We are now able to write the conservation law of a fractal function ε [9,10,11] into the so-called fractal space-time of its unchanged variant:

(11) $$\frac{\hat{d}\varepsilon }{dt}=\frac{\partial \varepsilon }{\partial t}+\mathrm{Vc}\cdot \nabla \varepsilon -i\frac{\lambda^{2}}{2\tau }{\left(\frac{dt}{\tau }\right)}^{\left(\frac{2}{D_{F}}\right)-1}\Delta \varepsilon =0$$

Segregating the two sides, respectively, the real part with respect to the imaginary part leads to

(12) $$\frac{\partial \varepsilon }{\partial t}+V\cdot \nabla \varepsilon =0,-U\cdot \nabla \varepsilon =\frac{\lambda^{2}}{\tau }{\left(\frac{dt}{\tau }\right)}^{\left(\frac{2}{D_{F}}\right)-1}\Delta \varepsilon$$

Hence, at the differentiable level, the topical variation in relation to time ∂ε/∂t and the term V⋅∇ε are equal, while at the nondifferentiable scale, the terms U⋅∇ε and Δε compensate each other.

In particular, for V=U (i.e., “synchronal” movements at differentiable and fractal scales), from Formula (12), we obtain the diffusion type equation

(13) $$\frac{\partial \varepsilon }{\partial t}=\frac{\lambda^{2}}{\tau }{\left(\frac{dt}{\tau }\right)}^{\left(\frac{2}{D_{F}}\right)-1}\Delta \varepsilon$$

Such a mathematical equation is involved by the Fourier heuristic model principle

(14) $$j(\varepsilon )=\frac{\lambda^{2}}{\tau }{\left(\frac{dt}{\tau }\right)}^{\left(\frac{2}{D_{F}}\right)-1}\nabla \varepsilon$$

where j(ε) is the current density. Therefore, Equations (13) and (14) describe the fractal fluid of conductive-type behavior [12,13].

In particular, for motions on fractal curves of the Peano’s type, i.e., in the fractal dimension $D_{F}\equiv 2$ [12,13,16], Formulas (13) and (14) take the standard forms

(15) $$\frac{\partial \varepsilon }{\partial t}=\frac{\lambda^{2}}{\tau }\Delta \varepsilon$$

and, respectively,

(16) $$j(\varepsilon )=\frac{\lambda^{2}}{\tau }\nabla \varepsilon$$

If ε is identified with the transient ionic current, I, then Equation (15) becomes

(17) $$\frac{\partial I}{\partial t}=\alpha \Delta I,\;\alpha =\frac{\lambda^{2}}{\tau }$$

In the one-dimensional case, the general solution is written in the form

(18) $$I=A+Bx+e^{i\omega t}\left[Ce^{\left(1+i\right)\sqrt{\frac{\omega }{2\alpha }}x}+De^{-\left(1+i\right)\sqrt{\frac{\omega }{2\alpha }}x}\right]$$

To establish the values of the constants *A*, *B* and *C*, it is necessary for *I* to be finite for *x = ∞*. In this case, *B* = *C* = 0, *A* = *I*_∞_ = *I*_0_ and

(19) $$I=I_{0}+e^{i\omega t}De^{-\left(1+i\right)\sqrt{\frac{\omega }{2\alpha }}x}=I_{0}+De^{-\sqrt{\frac{\omega }{2\alpha }}x}e^{i(\omega t-\sqrt{\frac{\omega }{2\alpha }}x)}$$

*Observation.* In the linear region, with the correlation between the current I and absolute temperature *T* being valid, we have the relation *I* = *mT* [10]. Furthermore, the plasma ion temperature *T_i_* is considered to be equal to the electron temperature (*T_e_*), assuming the local thermodynamic equilibrium (LTE) hypothesis.

However, because *I* is proportional to *T*, and making the following notations

(20) $$I=I_{0}+e^{i\omega t}De^{-\left(1+i\right)\sqrt{\frac{\omega }{2\alpha }}x}=I_{0}+De^{-\sqrt{\frac{\omega }{2\alpha }}x}e^{i(\omega t-\sqrt{\frac{\omega }{2\alpha }}x)}$$

we finally obtain

(21) $$T=T_{0}+\tilde{T}e^{-\frac{x}{\delta }}e^{i\omega (t-\frac{x}{v})},$$

of which the real part is equal to

(22) $$T=T_{0}+\tilde{T}e^{-\frac{x}{\delta }}\cos\omega \left(t-\frac{x}{v}\right)$$

Evidently, into a plane *x* = 0, where it is considered that the disturbance acts, we have

(23) $$T(0)=T_{0}+\tilde{T}\cos\omega t$$

In any case, from Formula (22), it results that the wave’s amplitude $\tilde{T}$ declines exponentially with the position in space (or the distance). We can now say that the fractal force *F*, in the conditions of a quasi-neutral plasma, is

(24) F=−∇Q

and *Q*, named the fractal potential, has the expression

(25) $$Q=-m\frac{U^{2}}{2}-m\frac{\lambda^{2}}{2\tau }\nabla \cdot U$$

and will only take the value zero [16]. In a favorable circumstance as this one, x=ct and Solution (22) describe waves, the amplitude of which exponentially decreases with time.

In this issue, a fractal pattern is an eloquent universality demonstration of self-organization processes produced in analyzed plasma discharge that is the result of an order of spontaneous symmetry breaking. In this philosophy, even the space-time concept is a fractal concept.

### 2.2. Theory versus Experimental Observations: Tsallis q-Statistics Interpretation

The classical principles of the scientific point of view in physical philosophy derive from the primacy of dynamics over statistics. This statement translates to the phrase “dynamics (the first) produces statistics” but not the other way around.

Nevertheless, to a complex model, their supposed holistic comportment does not easily allow such an abridgment and partition in statistics and/or fractal dynamics. The strange or bizarre kinetics, referring to both the dynamics and statistics stated above, can be considered two different images of one complicated construction and an integrative/exhaustive truth from the nonreductionist truth category [17,18].

In continuation of our exposure, we will introduce some theoretical assumptions about plasma. Additionally, we will make some necessary clarifications.

Firstly, we must mention here that we satisfy, in integrality, the physical significance of the Tsallis nonextensive entropy theory [18].

According to the status of the distribution function theory, the Tsallis q-triplet of nonextensive statistics will be utilized as experimental proof of q-statistics, as well as in fractal dynamics speculation. We must mention that through this applied research produced here, we will participate in a persuasive examination of the Tsallis theory in a complex dynamical system as determined by plasma plumes taken into account as equivalents.

**Figure 2:**
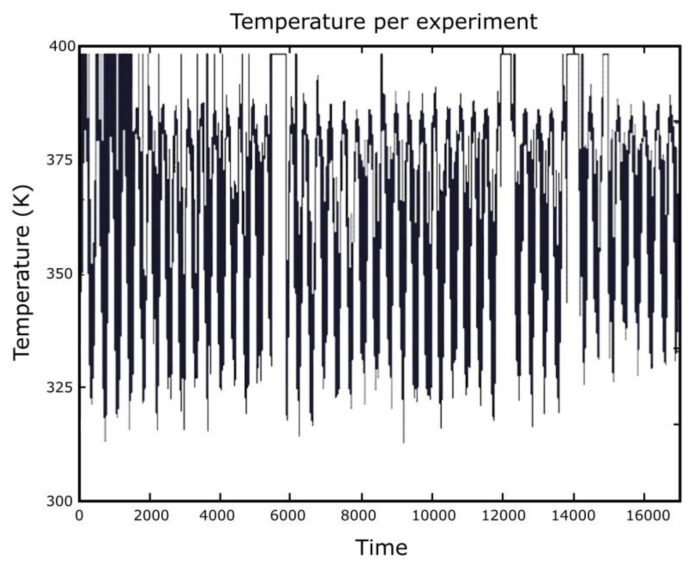
Temperature and function of time per experiment.

In Figure 2, a time series of absolute temperature (time-dependent temperature) is shown, and the superscript on the graph refers to the temperature per experiment. On the abscissa (*ox* axis), we have the time t (seconds), and on the ordinate (*oy* axis), we have the absolute temperature T (Kelvin).

The temperature was measured directly by a nanosecond time-resolution pyrometer, in fact by pyrometric thermal emission, which has evolved to determine the transient surface temperature of a material in the solid state of aggregation, heated by pulsed-excimer laser irradiation.

It is suggestive to memorize now that far from stability (out of equilibrium, as they say), the progress of spatiotemporal plasma structures through long-range correlations could be admissible. This was recommended by the assessment of Tsallis q-triplets (with nonzero values, found on review). Particularly, far from stability (far from the equilibrium point) the q-triplets family (q1, q2 and q3) can differ sufficiently from the equilibrium Gaussian-type contour (silhouette), where q1 = q2 = q3 = 1. By definition, these quantities are q1 = qsen = q-sensitivity (to initial conditions), q2 = qstat = q-statistics and q3 = qrel = q-relaxation (process), as presented in Reference [18].

Secondly, the Tsallis nonextensive entropy theory is associated with the multifractal and multiscale feature of the substantiating phase space, which, far from the stability condition (equilibrium), includes an abnormal topology related to multiscaling and multifractality characteristics.

**Figure 3:**
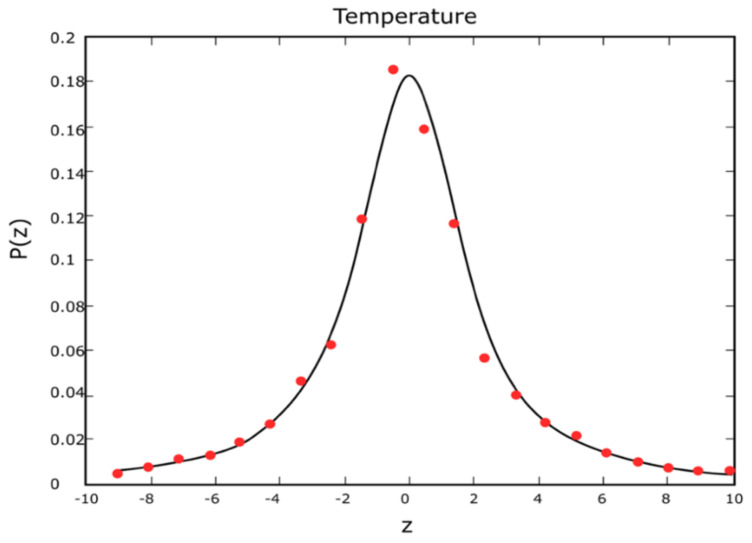
Probability repartition functions (PRFs). P(zi) versus zi into a q-Gaussian distribution function for adaptation of P(zi) at a given temperature.

Figure 3 refers to the determination of the normal or Gaussian distribution. Regarding the temperature (overwritten on the graphic representation), probability repartition functions (PRFs) P(z_i_) versus z_i_ for a q-Gaussian distribution function are shown (for adaptation of P(z_i_) at a given temperature).

This natural aspect is connected to the relevant multifractal reality and anomalous geometrical topology of the dispersal (dissipation) regions in the space-time physical theory. This seems to be the only logical conclusion.

Following this short philosophical speculation, we present proof of Tsallis nonextensive q-statistics for plume plasmas. Tsallis entropy, in connection with fractal dynamics and disorderly type mechanics of the plasma plume (chaotic regime) and time-series representation of plasmas temperature, is introduced for the first time in the present publication.

**Figure 4:**
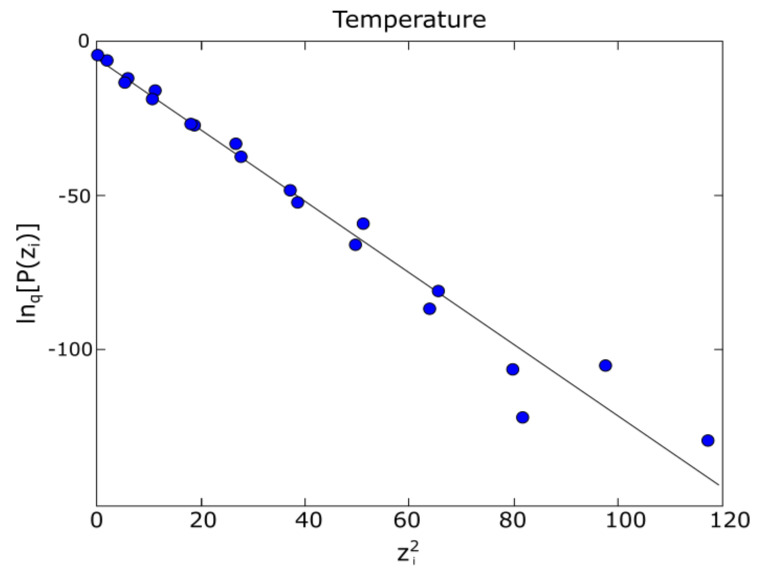
Graphic of ln_q_P(zi) versus (z_i_^2^) and its linear correlation at a given temperature.

Figure 4 refers to the establishment of linear correlation. Regarding the temperature (overwritten on the graphic representation), the linear correlation among ln_q_P(z_i_) and (z_i_^2^) is presented, more precisely, ln_q_P = ln_q_P(z_i_^2^), where q = 1.89 ± 0.08 for the temperature given.

At thermodynamic equilibrium, the underlying statistical dynamics are Gaussian (q = 1). As the system moves far from equilibrium, the underlying statistical dynamics become non-Gaussian; in other words, q is different from the value 1.

In conclusion, Figure 3 and Figure 4 graphically depict PRF P (z_i_) versus z_i_, in a q-Gaussian function (which accommodates P (z_i_) for the temperature given), and, respectively, linear correlation between ln_q_P (z_i_) and (z_i_^2^).

Since the Tsallis distribution is an extension of the Boltzmann–Gibbs distribution, it can be asserted that the thermal and mechanical characteristics of a complex system are a manifestation of the same natural process. This produces an identical thermal situation of the final physical state and at the mathematical extreme limit is dynamically associated orderly states (at the limit, Tsallis entropy is found, for example) [17,18]. According to this generic presentation in continuation, we provide a description of the concise justification of Tsallis nonextensive q-statistics for plume plasmas. The Tsallis distribution, in relation to the fractal dynamics and chaotic phenomena of the plasma area, will be introduced through a brief suite of phrases and mathematical expressions.

The *q_stat_* Tsallis index was evaluated by utilizing well-known probability repartition functions (*PRFs*), conformable to Tsallis q-exponential repartition:

(26) $$PRF\left[Z\right]=C_{1q}{{}^{\left[1+\left(q-1\right)C_{2q}{\left(\Delta Z\right)}^{2}\right]}}^{1/(1-q)}$$

where in the two coefficients *C*_1*q*_, *C*_2*q*_ denote the constants of normalization, and *q_stat_* # *q* (*q_stat_* is the same as *q* in Equation (26) above) is a nonextensivity element (named entropic factor) with respectto the relationship $q_{stat}\leq 3$, related to the queue dimension in the repartitions function. The obtained graphical histogram is rightly normalized, and the evaluated *q*-value conforms to the optimum linear adjustment for graphical representation of $\ln_{q}(p(z_{i}))$ (on the *oy* axis) depending on $z_{i}^{2}$ (on the *ox* axis). The obtained *q_stat_*, appropriate for optimum linear fitting, is then utilized to calculate the following formula:

(27) $$G_{q}(\beta ,z)=\frac{\sqrt{\beta }}{C_{q}}e_{q}^{-\beta z^{2}},\;\mathrm{where}\;C_{q}=\frac{\sqrt{\pi }\Gamma (\frac{3-q}{2(q-1)})}{\sqrt{q-1}\Gamma (\frac{1}{q-1})}$$

to various values of β and 1≤q≤3. Further, we select the ideal value of β, minimizing the relation

(28) $$\sum_{i}{G_{q_{stat}}(\beta ,z_{i})-p(z_{i})}^{2}$$

In principle, the following statistical investigation is supported in the algorithm depicted in [18]. In this mini-statistical subsection, the q-statistics related to the plasma plume temperature are studied.

The results are new, as we are the first authors to have applied the Tsallis statistical approach in analyzing the experimental data of a polymer plasma plume produced by laser ablation. This leads to a special understanding of the complex phenomenon in the space-time evolution of plasma plumes out of laser-produced plasma, and it anchors this phenomenon in the universality of none-equilibrium physical processes. First of all, it establishes a hierarchy between the types of statistics associated with the plasma plume of laser-produced plasma and occupies a special place alongside classic statistics, among which we list the one used mainly: Gaussian plume models.

We can compare our results with those obtained by Pavlos, but for space plasma. This type of plasma deals with an unlimited environment, unlike our environment, which is located in an isolated enclosure.

According to Nicolis, Prigogine [19], Hakens [20] and others, for physical systems far from equilibrium, new phenomena appear that are associated with global dynamics (long-range correlations). The system behavior far from equilibrium can be thought of as a phase transition in which the role of temperature corresponds to the progressive increase in nonequilibrium constraints [21]. These theoretical concepts were applied to physical space plasmas, where the complex system was modeled as an open, nonlinear and dissipative system.

### 2.3. Experimental Technique

In this subsection, we will discuss the experimental technique used. The investigational device is established on a structure that was initially used for analytical purposes and is described in detail in Reference [22].

**Figure 5:**
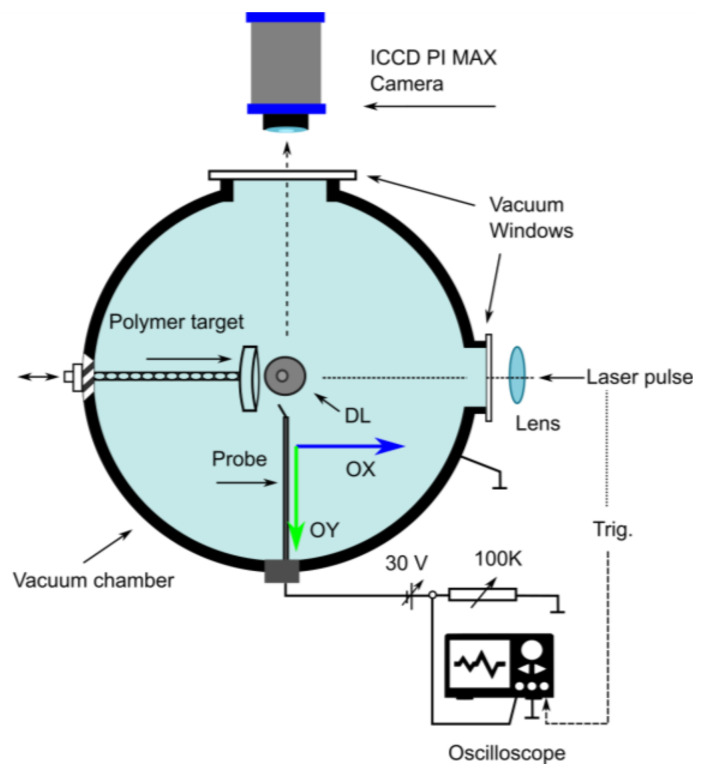
Presentation of the symbolic schema of the investigational setup.

Figure 5 shows the enclosure, including the vacuum chamber and the location of the polymer target and probe, together with the laser pulse and oscilloscope.

The tests were conducted in a vacuum chamber, built entirely of stainless steel and rapidly ejected by the ability of a 450 L/s turbo-molecular automatic pump device to a base pressure smaller than 10^−6^ torr (*p* < 10^−6^ Torr). As a necessary maneuver for the experimental process, it was then intervened by a 10 ns Nd: YAG laser and pulsed laser beam (with the wavelength λ = 532 nm), which was focused by a lens with focal radius f = 25 cm on the polymer target (the polymer composition is presented in the continuation of the paragraph) placed in the same enclosure as the vacuum chamber. The rough dot size in the percussion place was approximately 300 × 10^−6^ m (~300 µm).

The beam energy of the laser used (somewhere in between 1 and 100 × 10^−3^ J per pulse) was constantly supervised by the standardized high-resolution OPHIR PE10 joulemeter device (pyroelectric device). The energy currently engaged was ~40 × 10^−3^ J per pulse, which conducted to a classical laser intensity of ~5.7 × 10^9^ W/cm^2^.

We presented (also here) the experimental device used to explain the obtaining and behavior of the plasma plume at the same time as establishing the theoretical–experimental criteria, although we need it exhaustively in the second part of this study, called Part II: Experimental Results. This new part, focused on the processing of the experimental data obtained, will be the subject of an independent article that will be sent for publication later.

## 3. Results and Discussion

### 3.1. Observation on the Plasma Plume Expansion

The plasma plume dilatation was examined in a 2D coordinate system with distinctive normalized plane coordinates placed in the superior target area. More specifically, the *oy* axis concurs with the laser pulse symmetry axis, and the respective *ox* axis is arranged parallel to the target face/area.

In addition, the experimental results of a diblock copolymer film utilized as a target into the Nd: YAG laser (λ = 532 nm) ablation procedure were analyzed.

According to our device used, shown above, the exponential disintegrate coefficient has the value δ(t)=(4.16±0.34)MHz (with the frequency being 1 Hz = s^−1^), and the oscillation period is T=1/δ(t)=240 ns.

**Figure 6:**
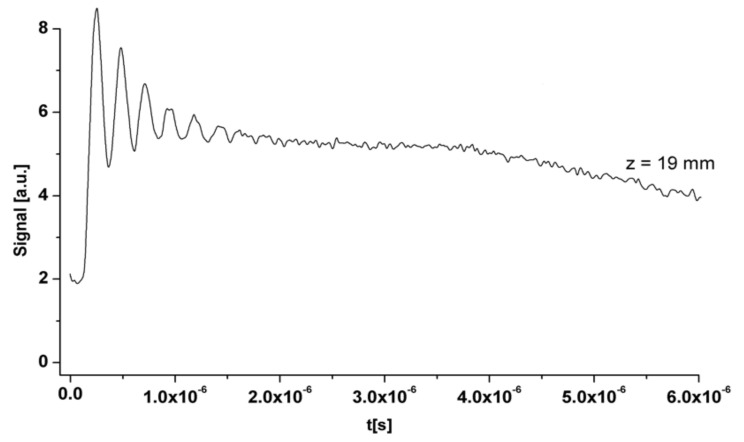
Oscillation of ionic signal (a.u.) versus time (s) (according to [20]).

In Figure 6, the transient ionic current, labeled signal on the graph, more precisely the transitory ionic signal (or ionic current intensity) registered by the Langmuir feeler/probe device placed at an established remoteness/length, perpendicular to the polymer target surface, is presented.

The currents induced in the polymer target by the laser ablation process can be associated with the probe device signal because the positive charge arises through the electrons escaping (a liberation action) from the expanding plasma plume in the electrically grounded chamber/enclosure, while the negative charge is given by the ions escaping from the target material support.

Again, considering that the correlation is valid in the linear region, we have the relation *I* = *aT*. The electron temperature (*T_e_*) is regarded to be equal to the plasma ion temperature (*T_i_*), in agreement with the local thermodynamic equilibrium (LTE) assumption valid under the given conditions.

As explained above, we have an oscillating behavior of temperature, which corresponds to the graph in Figure 2, analogous with the graph in Figure 6.

### 3.2. Temperature Estimation

**Figure 7:**
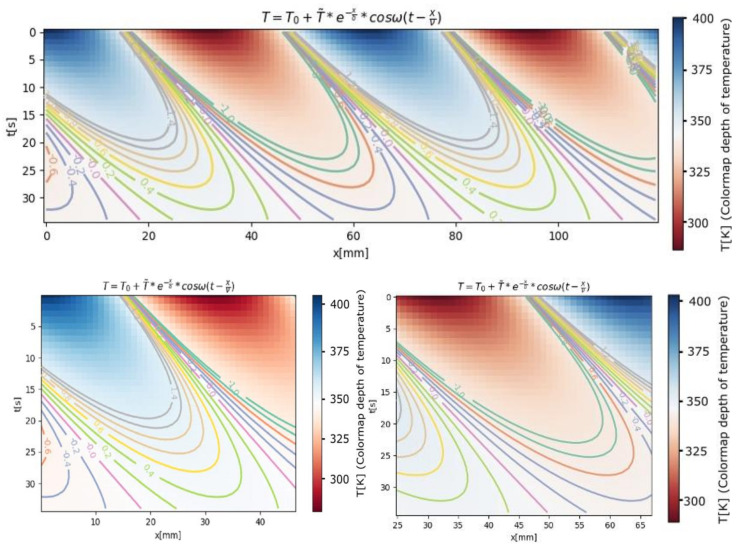
Two-dimensional (2D) time and position evolution of temperature. Application in colormap depth of temperature.

In Figure 7, the estimation of temperature versus *x*, position in the probe and time *t*, is shown. This is the graphical representation of the complete solution for the temperature distribution in a two-dimensional case.

We must mention that the complete solution for the temperature distribution and all graphic representations were obtained using the finite-differences method [23,24,25].

In the 2D graphs, the depth of temperature is specified by a colormap bar.

**Figure 8:**
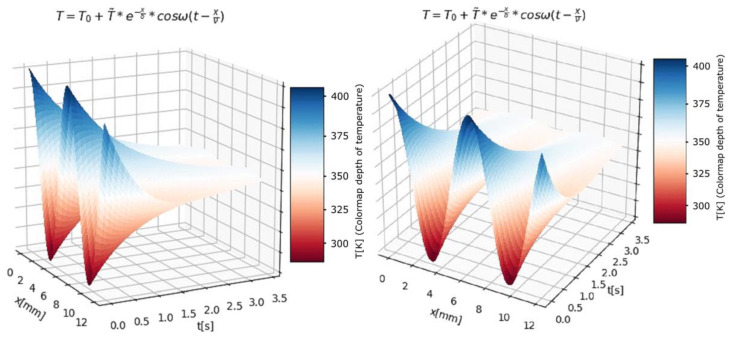
Three-dimensional (3D) graphs of temperature versus time and position.

The temperature is the same as that shown graphically in Figure 7 and Figure 8, where the absolute temperature varies between 300 K and 400 K. The temperature was measured electrically; more precisely, we measured the ionic current, and we took into account its proportional relationship with temperature.

In Figure 8, the estimation of the temperature function of *t* (time) and *x* (position in the probe) is presented. In the 3D graphs, the value of temperature is specified by a colormap bar.

## 4. Conclusions

Taking into consideration that the charge carrier travels are placed on nondifferentiable bends, to explain the electric charge transport in these conditions, a scale relativity suitable pattern was elaborated. Based on these considerations introduced by the authors, laser ablation and plasma plume generation were thus theoretically investigated. Regarding the fact that the charge carrier circulation is assumed as taking place on fractal curves, the so-called fractality, electrical charge transport can be resolved through a scale relativity extended method. A mathematical model, which is an original tool using the fractal space-time theory, was developed. Thanks to this theoretical evaluation of own system, polymer plasma plume dynamics, processed with the aid of laser ablation, were satisfactorily described. The obtained results fall successfully into the correct description of the behavior of polymer plasma feathers and allow understanding the research of this difficult field.

Tsallis entropy in direct connection with the fractal dynamics and chaotic-type mechanics of plasma plume and time-series representation of plasma temperature was introduced for the first time in the present publication by the authors. The theory was fully exploited using the appropriate concepts of probability physics. In a short statistical section, the q-statistics, as proof of Tsallis nonextensive q-statistics, for the plume plasma temperature were studied. The value obtained was *q* = 1.89 ± 0.08 for the temperature given. This aspect, where *q* is different from and higher than the value 1, means that the studied system in question moves far from equilibrium. In other words, the use of Tsallis entropy is well legitimized.

From the exploitation of the analytical model, the temperature function depending on two variables, time and position, was obtained. The graphics, obtained by the authors, related to representations of the complete solutions for the temperature distribution in two-dimensional and three-dimensional cases, were introduced.

All these observations made on polymeric plasma, obtained by means of laser ablation, complete the complex image of the phenomenon in question.

## Data Availability

The data used to support the findings of this study cannot be accessed due to commercial confidentiality.

## References

[1] Goldsten R.J., Rutherford P.H. (1995). Introduction to Plasma Physics.

[2] Weibel P., Ord G., Rössler G. (2005). Space-Time Physics and Fractality.

[3] Nottale L., Célérier M., Lehner T. (2006). Non-abelian gauge field theory in scale relativity. J. Math. Phys..

[4] Nottale L. (1993). Fractal Space-Time and Microphysics: Towards a Theory of Scale Relativity.

[5] Olteanu M., Paun V.-P., Tanase M. (2005). Fractal Analysis of Zircaloy-4 Fracture Surface. Rev. Chim..

[6] Paun V.P., Agop M., Chen G., Focsa C. (2018). Fractal-Type Dynamical Behaviors of Complex Systems. Complexity.

[7] Agop M., Paun V.P., Dandu-Bibire T. (2012). Chaos via Fractality in Gravitational Systems Dynamics. A New Approach (I). Int. J. Bifurc. Chaos.

[8] Ord G.N. (1983). Fractal space-time: A geometric analogue of relativistic quantum mechanics. J. Phys. A.

[9] Hauer M.R. (2004). Laser ablation of polymers studied by time resolved methods. Ph.D. Thesis.

[10] Gurlui S., Agop M., Nica P., Ziskind M., Focsa C. (2008). Experimental and theoretical investigations of a laser-produced aluminum plasma. Phys. Rev. E.

[11] Niculescu O., Dimitriu D.G., Paun V.-P., Matasaru P.D., Scurtu D., Agop M. (2010). Experimental and theoretical investigations of a plasma fireball dynamics. Phys. Plasmas.

[12] Camacho J.J., Oujja M., Sanz M., Martínez-Hernández A., Lopez-Quintas I., De Nalda R., Castillejo M. (2019). Imaging spectroscopy of Ag plasmas produced by infrared nanosecond laser ablation. J. Anal. At. Spectrom..

[13] Ravi-Kumara S., Liesa B., Lyub H., Qina H. (2019). Laser Ablation of Polymers: A Review. Procedia Manuf..

[14] Kigami J. (2001). Analysis on Fractals, Appendix B—Mathematical Background.

[15] Parvate A., Satin S., Gangal A.D. (2011). Calculus on Fractal Curves in R^n^. Fractals.

[16] Ioannou P.D., Nica P., Paun V., Vizureanu P., Agop M. (2008). Wave-particle duality through an extended model of the scale relativity theory. Phys. Scr..

[17] Ferri G.L., Reynoso Savio M.F., Plastino A. (2010). Tsallis q-triplet and the ozone layer. Physic A.

[18] Pavlos G.P., Xenakis M.N., Karakatsanis L.P., Iliopoulos A.C., Pavlos A.E.G., Sarafopoulos D.V. (2012). Universality of Tsallis Non-Extensive Statistics and Fractal Dynamics for Complex Systems. Chaotic Model. Simul. (CMSIM).

[19] Prigogine I., Nicolis G. (1967). On Symmetry-Breaking Instabilities in Dissipative Systems. J. Chem. Phys..

[20] Haken H. (1975). Cooperative Phenomena in Systems Far from Thermal Equilibrium and in Nonphysical Systems. Rev. Mod. Phys..

[21] Pavlos G.P., Iliopoulos A.C., Athanasiou M.A., Karakatsanis L.P., Tsoutsouras V.G., Sarris E.T., Kyriakou G.A., Rigas A.G., Sarafopoulos D.V., Anagnostopoulos G.C. (2011). Complexity in Space Plasmas: Universality of Non-Equilibrium Physical Processes. AIP Conf. Proc..

[22] Niculescu O., Petre Nica P., Gurlui S., Forna N., Casian-Botez I., Ionita I., Constantin B., Badarau G. (2009). Experimental Investigations of Polymer Plasma Laser Ablation. Mater. Plast..

[23] Zienkiewicz O.C., Taylor R.L., Zhu J.Z. (2005). The Finite Element Method: Its Basis and Fundamentals.

[24] Fish J., Belytschko T. (2007). A First Course in Finite Elements.

[25] Rao S.S. (2020). The Finite Element Method in Engineering.

